# Tubocutaneous Fistula

**DOI:** 10.1155/2015/104360

**Published:** 2015-04-07

**Authors:** Krishnaveni Nayini, Clive Gie

**Affiliations:** Department of Obstetrics and Gynaecology, King's Mill Hospital, Sutton-in-Ashfield, Nottinghamshire NG17 4JL, UK

## Abstract

*Introduction*. Tubocutaneous fistula is a very rare condition; most cases described in the literature are secondary to endometriosis, tuberculosis, and complications of child birth and gynecological operations. *Case Presentation*. We report a case of 40-year-old woman who presented with tubocutaneous fistula secondary to pelvic inflammatory disease which was diagnosed in the setting of persistent discharging wound in the right groin. *Conclusion*. Tubocutaneous fistula is a rare condition. Salpingectomy and resection of fistulous tract is the treatment of choice as is treating the underlying cause. Early diagnosis and treatment of these patients are essential for avoiding long term complications.

## 1. Introduction

Gynecologists are familiar with the vesicovaginal, uretrovaginal, and rectovaginal fistulae [1]. There are also case reports regarding uterocutaneous and salpingo enteric fistulae in literature. There are no cases reported regarding tubocutaneous fistula. This is a rare case of tubocutaneous fistula which developed after treating the pelvic abscess. The fistula extended from the right fallopian tube to the right groin along the anatomical path of the round ligament.

## 2. Case Presentation

A 40-year-old woman with 2 previous normal deliveries presented to surgeons at King's Mill Hospital in April 2013 with a 3-year history of intermittently discharging persistent right groin sinus. The discharge was purulent in nature; she often felt generally unwell. The patient had a past medical history of laparotomy for ruptured pyosalpinx in 2000. Subsequently the patient was seen in gynecology clinic with a history of recurrent lower abdominal pain in 2008 and 2011. Initially the patient was treated with antibiotics and she was given the option of pelvic clearance. The patient elected to have conservative management.

On examination by the surgeons the patient was noted to have a discharging sinus in the right groin with the working diagnosis of hidradenitis. A CT sinogram was organized. The sinogram demonstrated a sinus extending from the right groin crease to the right adnexa with the contrast tracking behind the uterus and the upper vagina. In view of these findings the patient was referred to the gynecology team for further assessment.

At gynecological assessment the patient reported a history of right iliac fossa pain and constant discharge from the right groin sinus. On abdominal examination the patient was noted to have a midline laparotomy scar and discharging right groin sinus. On pelvic examination there was reduced mobility of the pelvic organs and thickening noted in the right adnexal region. After liaising with the surgeons a plan was made for laparotomy, right salpingo oophorectomy, and excision of fistulous tract. The patient was informed regarding the risks to adjacent vascular structures due to the proximity of the tract to the major blood vessels.

The patient had a laparotomy under the joint care of the gynecologists and surgeons on May 27, 2014. At laparotomy the patient was noted to have a normal left tube and ovary which was attached to left pelvic side wall. The uterus was normal and she had a normal right ovary and right pyosalpinx. The fistulous tract was communicating to the cornual end of right fallopian tube posterior to the round ligament; it then followed the path of the round ligament, opening at the level of inguinal ring in the right groin.

Right salpingectomy was performed and the right round ligament was divided near the right cornual end of the uterus; a right groin fistulous tract of 3 cm was excised. Histopathological examination demonstrated pyosalpinx with features of chronic and acute inflammation with microabscesses and fistulous tract showing inflammatory granulation tissue without evidence of malignancy. The patient was discharged home on the 3rd postoperative day.

The patient was readmitted on the 6th postoperative day with headache. She was seen by the anesthetists who treated her with a blood patch for postdural headache. The patient made a good postoperative recovery followed by the blood patch and she was discharged home on the 8th postoperative day. The patient was reviewed in the clinic at 8 weeks postoperatively. She had made a good recovery with completely healed right groin wound and laparotomy scar.

## 3. Discussion

The communication between the fallopian tube and skin is very rare. This communication can result from obstetric, surgical, and medical complications such as pelvic inflammatory disease, endometriosis, tuberculosis, pelvic irradiation, inflammatory bowel disease, and pelvic surgery [2].

In our case the fistulous tract was following the path of the round ligament and it has developed after recurrent episodes of pelvic inflammatory disease. HSG, ultrasound, CT, and MRI are the investigation modalities of choice. CT scan is proved to be superior as it gives information regarding etiology of fistula and extent of extraluminal disease [3, 4].

The treatment depends on the age of the patient and the fertility options of the women. Fistulas to the fallopian tubes are rare and their management is not very well described. Most studies advocate fistula resection and salpingectomy as the only feasible method of treating this rare disease to prevent the occurrence of ectopic pregnancy subsequently [3]. Those resulting from Crohn's disease and complicated diverticulitis, en-bloc fistula resection, and salpingectomy are also recommended. However, the type of resection will be tailored to the needs of the patient.

## 4. Conclusion

Tubocutaneous fistulae are rare; their management is not well described in the literature. Salpingectomy, resection of fistulous tract, and treating the underlying cause are the treatment of choice. Early diagnosis and treatment of these patients are essential for avoiding long term complications.

## Abbreviations

CT: Computed tomography
HSG: Hysterosalpingogram
MRI: Magnetic resonance imaging.
